# Recovery of 2,3-Butanediol from Fermentation Broth by Zeolitic Imidazolate Frameworks

**DOI:** 10.1021/acs.iecr.3c01925

**Published:** 2023-10-03

**Authors:** Yadong Chiang, Qiang Fu, Wanwen Liang, Arvind Ganesan, Sankar Nair

**Affiliations:** † School of Chemical & Biomolecular Engineering, Georgia Institute of Technology, 311 Ferst Drive NW, Atlanta, Georgia 30332-0100, United States; ‡ School of Chemistry and Chemical Engineering, South China University of Technology, Tianhe District Wushan Road Number 381, Guangzhou, 510640, China

## Abstract

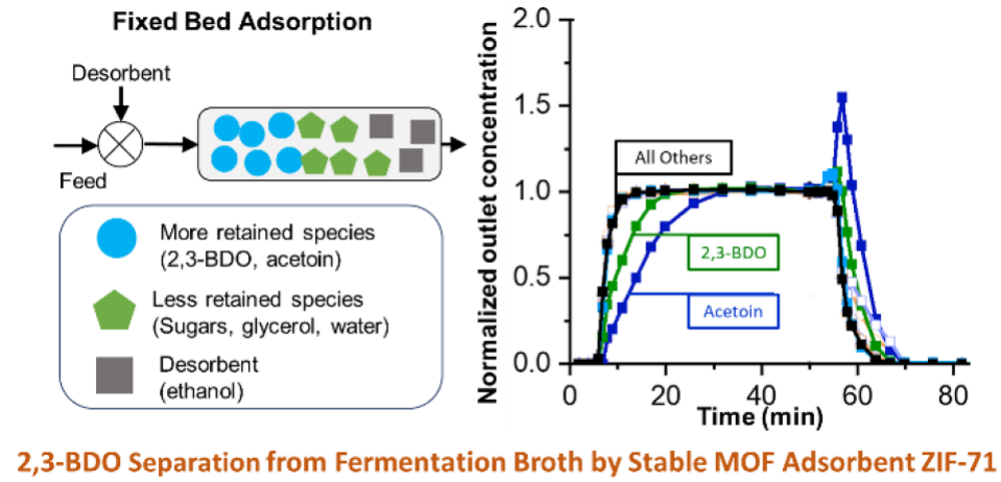

The efficient separation of the 2,3-butanediol (2,3-BDO) intermediate from fermentation broth is an important issue in the production of biofuels from biomass-derived intermediates. Two zeolitic imidazolate frameworks ZIF-8 and ZIF-71 were investigated for the adsorption of 2,3-butanediol (2,3-BDO) from fermentation broth via liquid breakthrough adsorption measurements. While both ZIF materials initially show high separation performance, ZIF-71 retains robust separation performance even after aging in ethanol for two years, whereas the capacity of ZIF-8 decreases significantly. The robustness and stability of ZIF-71 are further confirmed with cyclic fixed bed adsorption measurements. The uptake of 2,3-BDO on ZIF-71 reaches >100 g/kg with negligible uptakes of sugars, organic acids, and other alcohols present in the fermentation broth. Excellent selectivity toward 2,3-BDO over water is also achieved. The 2,3-BDO-loaded ZIF-71 can be regenerated efficiently with ethanol as desorbent. These findings indicate that ZIF-71 shows considerable promise as an adsorbent to recover and purify diols from fermentation broths.

## Introduction

Production of biomass-derived aviation fuels and diesel is of great interest due to the emphasis on decarbonized fuel and chemical production.^1−3^ Typically, biomass is first converted to produce intermediates that are catalytically converted to hydrocarbon fuels. Among such intermediates, 2,3-butanediol (2,3-BDO) has emerged as one of the most important. Its C_4_ size confers advantages over C_1_ and C_2_ intermediates during catalytic conversion to liquid hydrocarbon mixtures, and it also has many other applications as a platform chemical.^4−10^ The biological (fermentative) production of 2,3-BDO has made considerable progress with a particularly attractive route starting from lignocellulosic wastes (e.g., corn stover).^11,12^ However, the high boiling point (180 °C), high affinity with water, and low concentration (∼10 wt %) in typical fermentation product broths create a significant challenge in 2,3-BDO separation from the complex fermentation broths that also contain residual sugars, fermentation byproducts, and biological macromolecules. This issue becomes a significant bottleneck for the development of economical processes for biofuel production via the 2,3-BDO route.^13^

Several separation technologies have been proposed, such as steam stripping, solvent extraction, pervaporation, reactive extraction, salting-out extraction, and adsorption.^14−21^ Selective adsorption in a nanoporous material, when coupled with a volatile (easily regenerated) desorbent, is an attractive and energy-efficient separation route for 2,3-BDO. The adsorption of 2,3-BDO has been little explored. ZSM-5 zeolite allowed 2,3-BDO uptake of ∼45 g/kg from a synthetic broth, which increased to ∼59 g/kg upon modification of the zeolite surface with fluorocarbon groups.^19^ The affinity between organic adsorbates and the adsorbent is one of the key aspects of adsorbent selection. Table S1 (Supporting Information) depicts the hydrophobicity of the significant molecules present in currently available 2,3-BDO fermentation broths using the partition coefficients (*K*_ow_). Their molecular sizes are indicated by the kinetic diameter (KD). Sugars and polyols possess larger kinetic diameters and hydrophilic features, whereas diols (2,3-BDO), acetoin, ethanol, and organic acids are relatively hydrophobic and have small kinetic diameters. Acetoin (3-hydroxybutanone) is chemically similar to 2,3-BDO and can be catalytically coprocessed; hence, it is also desired to be recovered along with 2,3-BDO. We hypothesized that 2,3-BDO (and acetoin) can be recovered and separated from sugars and polyols, as well as from the other smaller molecules, by nanoporous materials with a combination of hydrophobicity and appropriate pore sizes. Furthermore, we hypothesized that certain types of metal organic frameworks (MOFs) could provide higher porosity and tunable functionality over other nanoporous materials such as zeolites. Zeolitic imidazolate frameworks (ZIFs), a subset of MOFs, consist of tetrahedral metal ions (e.g., Zn, Co) bridged by four imidazolate ligands in the same way as Si and Al atoms are coordinated by bridging oxygens in zeolites.^22^ For example, three ZIF materials have previously been studied for adsorption of 5-hydroxymethylfurfural (HMF) from an aqueous solution, with ZIF-8 [Zn(2-methylimidazole)_2_] seen to provide the best adsorption performance due to its highest hydrophobicity.^23^ On the other hand, we have recently shown that the RHO topology ZIF-71 (containing 4,5-dichloroimidazole linkers) possesses remarkable water and humid acid gas stability among ZIF materials.^24^ However, the applications of ZIF-71 in liquid adsorption have been little-explored. In this work, we investigated ZIF-8 and ZIF-71 as adsorbents for the recovery of 2,3-BDO from both model and actual fermentation broths. Adsorption experiments were performed through freshly prepared packed bed columns and compared with the performance after aging in ethanol for two years. The performance and stability were further investigated through cyclic adsorption measurements.

## Materials and Methods

Experimental details are provided in the Supporting Information, including the materials synthesis and characterization, adsorption column preparation, description of breakthrough measurements, and samples analysis methods. The relevant literature references (23, 25, 26) are also cited therein.

## Results and Discussion

Among the multiple synthesis procedures available for ZIF-8 and ZIF-71, methods were chosen to produce adsorbents in which the primary particles are below 100 nm. This allowed us to eliminate intraparticle mass transfer limitations. The morphology of the synthesized ZIF-8 and ZIF-71 adsorbents are shown by the SEM images in Figures S1a,b. Both adsorbents display primary nanoparticle sizes in the 50–100 nm range, and these nanoparticles are aggregated together to form larger adsorbent particles. These particles are further used for production of adsorbent pellets (see Supporting Information), which are packed into columns. The crystallinity and porosity of the pelletized adsorbents were examined by using XRD and N_2_ physisorption. Figure S2 confirms the high X-ray crystallinity of both materials, and the N_2_ physisorption isotherms shown in Figure S3 confirm that the adsorption behavior for both adsorbents is Type I (micropore adsorption). The BET surface area and micropore volume are also calculated from the isotherms (Table S2) and conform to the typical textural characteristics of the ZIF-8 and ZIF-71 materials. ZIF-8 possesses a larger BET surface area and micropore volume but has a smaller limiting pore size.

The ZIF-8 and ZIF-71 adsorbent columns were used for liquid breakthrough experiments to examine the equilibrium separation and adsorption kinetics for the separation of 2,3-BDO from a simplified model broth mixture. The model broth (see Table 1) consists of sugars like xylose and glucose (representing the unreacted feedstock), glycerol, and acetoin (byproducts), and 2,3-BDO as the primary product, at concentrations similar to those in the real fermentation product broth. Measurements were made on the freshly packed columns and then repeated after two years of storage under ethanol (which is used as a desorbent). In these experiments, adsorption is performed by feeding the model broth for 360 min into the packed columns presaturated with the desorbent (i.e., ethanol). The separation characteristics of the freshly packed columns are shown by the breakthrough curves in Figures S6a,b. The outlet concentration of each component is normalized by its concentration in the feed. Sugars like glucose and xylose break through early since they are both bulky in size, and thus, their rejection by the ZIF adsorbents is dominated by size-exclusion mechanisms. Water and glycerol break through almost immediately after glucose and xylose. Given their relatively smaller kinetic diameters (KDs), the rejection of these components (especially water) is clearly dominated by the high hydrophobicity of the adsorbents. The 2,3-BDO and acetoin (which have a nearly identical structure) are adsorbed by both ZIF-8 and ZIF-71. Overall, the order of breakthrough indicates their relative selectivities. The nonmonotonic concentration profiles for 2,3-BDO and acetoin indicate some influence of diffusion limitations in the freshly prepared columns. In Figure S6b (ZIF-71), both 2,3-BDO and acetoin exhibit an early onset of breakthrough, followed by a drop of outlet concentrations between 5 and 10 min, and then a slow increase thereafter approaching equilibrium. These characteristics are attributed to slow diffusion of these molecules in the two ZIF adsorbents as well as competitive adsorption (acetoin has a stronger adsorption strength on the two ZIF materials and displaces the adsorbed 2,3-BDO). Irrespective of the kinetic behavior, the equilibrium uptakes of each component and the separation factor of the 2,3-BDO/water pair can be calculated based on the breakthrough curves, as shown in Table 1. For the freshly packed columns, adsorption of 2,3-BDO and acetoin are significant while xylose and glycerol are negligibly adsorbed. A significant amount of water (albeit much lower than in the liquid feed) is also observed in the equilibrium adsorbed phase, due to is already high concentration (∼90 wt %) in the liquid phase. Both adsorbents show similar and very high (>70) separation factors for the 2,3-BDO/water pair and also for 2,3-BDO and acetoin over all the other organics. However, the uptake of 2,3-BDO on ZIF-8 is considerably higher due to its higher micropore surface area and micropore volume.

**Table 1:** Composition of Model Broth and Adsorption Uptake of Aged Columns after Two Years (with Data from Freshly Packed Columns in Parentheses)

		Equilibrium Uptake (g/kg adsorbent)	
Component	Feed Conc. (g/L)	ZIF-8	ZIF-71
Glucose^a^	0.2	/	/
Xylose	8.1	3.5 (0.1)	0.5 (0.4)
Glycerol	3.6	1.1 (0.5)	0.8 (0.5)
Acetoin	8.6	20.5 (83.2)	51.0 (43.4)
2,3-BDO	64	47.0 (83.4)	109.6 (50.3)
Water	930	376.1 (15.9)	25.8 (10.1)
*S*_2,3-BDO/Water_		2 (76)	62 (72)

a Glucose is regarded as non-adsorbing tracer.

**Figure 1:**
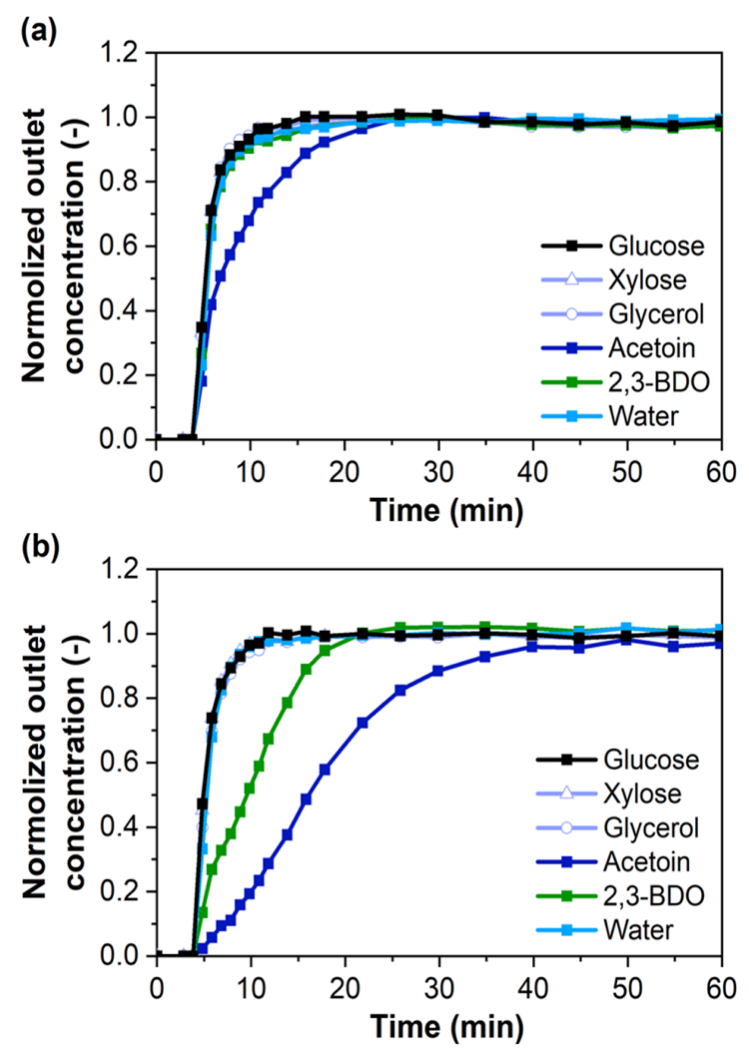
Multicomponent liquid (model broth) breakthrough adsorption at 303 K using regenerated (a) ZIF-8 and (b) ZIF-71 columns, two years after packing.

Both ZIF-8 and ZIF-71 columns were then regenerated with 0.2 mL/min ethanol desorbent for 360 min. The columns were then closed and stored/aged *in situ* under ethanol for two years. Figures 1a,b show the adsorption breakthrough curves on the same columns using the same model broth formulation after two years of adsorbent storage. As compared with the freshly packed columns, adsorption behaviors on both columns are different after two-year storage, especially the concentration profiles of 2,3-BDO and acetoin. Unlike the nonmonotonic concentration profiles for these two components in the freshly packed columns (Figures S6a,b), the regenerated and aged columns show monotonic behavior (Figure 1). In the case of ZIF-8 (Figure 1a), the breakthrough of all the components is almost simultaneous with only acetoin showing a significantly delayed breakthrough, thus indicating significant degradation of the adsorbent function upon aging of the column. However, in the ZIF-71 column (Figure 1b), the breakthrough of 2,3-BDO and acetoin remains much slower than that of the other components and continues to show strong separation of these components from water and the other organics. In fact, the breakthroughs of 2,3-BDO and acetoin are slower in Figure 1b than in Figure S6b, indicating that the aged ZIF-71 column does not display diffusion limitations.

The changes in the adsorption performance of ZIF-8 and ZIF-71 columns upon aging are quantified in Table 1. On ZIF-8, the uptake of the desired components (2,3-BDO and acetoin) is dramatically decreased, while those of water, xylose, and glycerol are increased. The large increase in water uptake after aging, even though the column was stored in ethanol (and not water), indicates the loss of hydrophobicity of ZIF-8. It is also consistent with the decrease of selectivity for 2,3-BDO over water. In the case of ZIF-71, the uptakes of 2,3-BDO and acetoin increase dramatically after aging. The uptakes of water, xylose, and glycerol also increase moderately. Overall, the selectivity between 2,3-BDO and water is retained. Both the aged ZIF-8 and ZIF-71 were removed from the column and characterized. In ZIF-8, the large increase in water uptake and the loss of 2,3-BDO/water separation factor (i.e., loss of hydrophobicity) are very likely due to defects created by slow breakage of the metal (Zn)-linker (2-methylimidazole) coordination bonds, that are slowly replaced by Zn–OH and protonated linker molecules. This is indicated by the dramatically increased oxygen content in the aged ZIF-8 relative to that in pristine ZIF-8 as seen from XPS data (Table S3 and Figure S4a). The nominal oxygen content detected in pristine ZIFs is attributed to the termination defects present on the surface of the ZIF crystals. Given the small particle sizes (high external surface area) and low penetration depths of XPS scans, some oxygen content is anticipated.^27^ These observations are also supported by water vapor adsorption measurements (Figure S5). In aged ZIF-8, the water uptake capacity increases by ∼300% relative to pristine ZIF-8, due to the introduction of Zn–OH defects during aging. The crystal morphology of ZIF-8 also became larger and more disordered (Figures S1a,c). Figure S2a (XRD patterns) shows the increased framework disorder of ZIF-8 upon aging, with the upward shift in baseline.^28,29^ This also leads to a decrease in the pore volume and surface area seen in Table S2.

**Figure 2:**
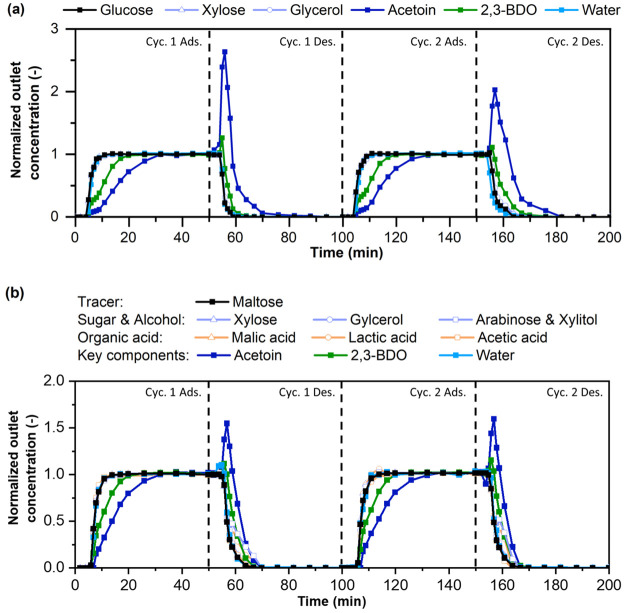
Exit concentration profiles from two adsorption/desorption cycles over the aged ZIF-71 column with two different feeds: (a) model broth and (b) actual fermentation product broth. Profiles are expressed on a desorbent-free (i.e., ethanol-free) basis.

In ZIF-71, the breakage of the metal-linker coordination bond may occur at a much slower rate. It has recently been shown that ZIF-71 has exceptional stability toward metal-linker coordination bond breakage, whereas ZIF-8 does not.^24^ As a result, in the present case, the low density of defects (missing linkers) may be able to accelerate diffusion by effectively increasing the limiting pore size upon removal of some of the bulky linkers (4,5-dichloroimidazole), consistent with the 2,3-BDO and acetoin breakthrough profiles in Figure 1b compared with Figure S4b. On the other hand, unlike ZIF-8, the crystal size of ZIF-71 remains uniform after aging (Figure S1d and Figure S1a), which is consistent with the XRD patterns (Figure S2b). The smaller and more uniform crystal size also facilitates diffusion in ZIF-71 after aging. Furthermore, this process also appears to significantly increase the available pore volume and surface area (as indicated by Table S2) for adsorption of 2,3-BDO and acetoin (as evinced by the uptakes shown in Table 1) while maintaining a low uptake of water. The high hydrophobicity is seen from the insignificant change of water uptake in Figure S5 and the oxygen content in Table S3 and Figure S4b, between the pristine and aged ZIF-71. We note that the oxygen content in pristine ZIF-71 is higher than pristine ZIF-8, because the concentration of termination defects is affected by several structural and synthesis parameters such as the topology, metal concentration on the surface of crystal, etc. Hence, controlled aging of ZIF-71 under ethanol allows excellent performance including 2,3-BDO uptake >100 g/kg adsorbent as well as high 2,3-BDO/water selectivity >60, whereas ZIF-8 loses 2,3-BDO/water selectivity and becomes highly hydrophilic. Comparison of the freshly packed columns alone would lead to an incorrect selection of ZIF-8 as a preferred adsorbent for 2,3-BDO recovery. Based on the above findings, we chose the aged ZIF-71 packed column to perform two adsorption/desorption cycles at 303 K with the model broth (Figure 2a) and then the actual fermentation product broth (Figure 2b) that was produced by an established process.^26^ Ethanol was used as the desorbent. The outlet concentrations of components were measured as a function of time during the cycle and are normalized by the corresponding feed concentration (Table 1). See Supporting Information for further details of adsorption/desorption cycling measurements and pretreatment of the fermentation product broth.

The adsorption/desorption behavior of the ZIF-71 column for the model broth feed is shown in Figure 2a. The concentration profiles during the adsorption stages of both cycles are consistent with those in Figure 1b. In the corresponding desorption stages (using ethanol as desorbent), the exit composition of the initial ∼5 min (on ethanol-free basis) is the same as the feed, since the desorbent is initially displacing the aqueous feed present in the interstitial spaces between the adsorbent particles. Once the aqueous phase is displaced from the column, desorption peaks of the adsorbed components is seen. The desorbent outlet stream after 30 min of desorption has negligible adsorbed components, indicating that ethanol is an effective desorbent. Figure 2b shows the cycling behavior with the real fermentation product broth, which contains sugars, alcohols, organic acids, 2,3-BDO, and acetoin (which has a much lower concentration in the real broth than the model broth; see Table 2). The adsorption of 2,3-BDO and acetoin follows the same profiles as in the model broth (Figure 2a). All the other components break through immediately and are not adsorbed, highlighting the remarkable selectivity of the ZIF-71 adsorbent in capturing 2,3-BDO and acetoin. Additionally, desorption of 2,3-BDO and acetoin proceeds in a similar manner as in the model broth, with only slight differences in the profiles. The column can be fully regenerated with ethanol at a desorbent-to-feed ratio (D/F) < 1, which indicates the ease of regeneration of the ZIF-71 column. In the case of the real broth, we did not observe any deleterious effects on the column caused by the broth components e.g., potential precipitation of sugars in ethanol or fouling of the adsorbent from trace/unknown contaminants in the broth. Table 2 shows the composition of the real fermentation broth, the uptakes of each component, and the 2,3-BDO/water selectivity of the ZIF-71 column from the real fermentation broth (the corresponding data for the model broth are shown in parentheses). Based on these data, the 2,3-BDO purity (on desorbent-free basis) in the extract product is ∼65% (averaged between the two cycles). This is a large enhancement from the 9.9% concentration of 2,3-BDO in the fermented broth feed. In a single pass, ∼93% of the water is separated from the 2,3-BDO product. These characteristics indicate that ZIF-71 is a robust and promising adsorbent to recover and purify 2,3-BDO from actual fermentation broth. The separation factor of 2,3-BDO/water is high, specifically, ∼17 (average over two cycles). The difference in the separation factor between the model and real broth cases is mostly due to the nonlinearity of the 2,3-BDO adsorption isotherm. Due to the considerably higher concentration of 2,3-BDO in the real broth than in the model broth, the ratio of the adsorbed mass uptake and the concentration becomes smaller.

**Table 2:** Cyclic Adsorption Data on the ZIF-71 Column Using Actual Fermentation Broth^a^

		Uptake (g/kg ZIF-71)	
	Feed Concentration (g/L)	Cycle 1	Cycle 2
Glucose^b^	- (0.2)	/	/
Maltose^b^	8.2 (−)	/	/
Xylose	2.7 (8.1)	0.0 (0.7)	0.0 (0.5)
Glycerol	7.3 (3.6)	0.1 (0.8)	0.0 (0.8)
Arabinose and Xylitol^c^	7.7 (−)	0.3 (−)	0.0 (−)
Malic acid	2.5 (−)	0.0 (−)	0.0 (−)
Lactic acid	2.5 (−)	0.0 (−)	0.0 (−)
Acetic acid	2 (−)	0.2 (−)	0.6 (−)
Acetoin	0.7 (8.6)	2.3 (42.8)	2.4 (39.6)
2,3-BDO	101.1 (64)	129.4 (142.6)	124.2 (130.7)
Water	889 (930)	58.4 (38.4)	67.8 (49.3)
*S*_2,3-BDO/Water_		19 (54)	16 (38)

a Data from the Model Broth Are Shown in Parentheses.

b Glucose and maltose are regarded as non-adsorbing tracers.

c Total concentration of arabinose + xylitol is calculated together due to overlapping peaks in HPLC analysis.

## Conclusions

The selective recovery of 2,3-BDO and acetoin from aqueous mixtures has been investigated on the two zeolitic imidazolate frameworks ZIF-8 and ZIF-71 via packed-bed multicomponent breakthrough measurements. In freshly packed columns, both hydrophobic ZIF materials show similar high performance for separation of 2,3-BDO and acetoin from water and the other organic components, with ZIF-8 showing higher uptake of 2,3-BDO due to a larger internal surface area. However, upon aging the two columns in ethanol (which is used as a desorbent) for two years, the adsorption behavior changes dramatically. ZIF-8 loses 50% adsorption capacity and nearly all selectivity for 2,3-BDO recovery. However, ZIF-71 shows a dramatic (100%) increase in the 2,3-BDO adsorption capacity and also maintains selectivity. These results indicate that stable MOFs such as ZIF-71 can be applied as adsorbents to selectively recover organic molecules (such as 2,3-BDO and acetoin) from complex aqueous mixtures (such as fermentation broths). The specific mechanistic aspects of this aging process and its impact on the adsorption performance are not yet known, but the present results are consistent with recent findings on the remarkable chemical stability of ZIF-71 relative to those of other members of the ZIF family.

## Supporting Information Available

Detailed description of materials, methods, and mathematical formulas; Tables S1–S3 containing detailed data on feed component properties and structural characteristics of the ZIF adsorbents; and Figures S1–S6 depicting structural and separation characteristics of the ZIF materials (PDF)
