# Supplementary material for: Recovery of 2,3-Butanediol from Fermentation Broth by Zeolitic Imidazolate Frameworks
Source: Ind Eng Chem Res. 2023 Oct 3;62(41):16939–44. doi: 10.1021/acs.iecr.3c01925 (PMC10588442; doi:10.1021/acs.iecr.3c01925)
Supplement: Supplementary file 1 — ie3c01925_si_001.pdf [file ie3c01925_si_001.pdf]

Supporting Information

for

Recovery of 2,3-Butanediol from Fermentation Broth by Zeolitic Imidazolate Frameworks

Yadong Chiang[1†], Qiang Fu[1†], Wanwen Liang[1,2], Arvind Ganesan, and Sankar Nair[1]*

[1]*School of Chemical & Biomolecular Engineering, Georgia Institute of Technology*

311 Ferst Drive NW, Atlanta, GA 30332-0100, USA

[2]*School of Chemistry and Chemical Engineering, South China University of Technology*

Tianhe district, Wushan Road, Number 381, Guangzhou, China 510640

Corresponding Author

*Email: sankar.nair@chbe.gatech.edu

† Equal contribution

Number of pages: 12

Number of supporting tables: 3

Number of supporting figures: 6

Materials and Methods

Materials

Chemicals used in ZIF material synthesis (zinc nitrate hexahydrate, zinc acetate, 2-methylimidazole, and 4,5-dichloroimidazole) were purchased from Sigma-Aldrich. Chemicals used to prepare model broths, such as 2,3-butanediol (2,3-BDO, mixture of racemic and meso forms), D(+)-glucose, D(+)-xylose, arabinose, malic acid, and xylitol were purchased from Acros Organics. Lactic acid, acetic acid, glycerol, and ethanol were purchased from Fisher Chemical. Maltose was purchased from Fisher BioReagents. All the above chemicals were used as received.

Pretreatment of fermentation broth

The fermentation product broth was received from Oak Ridge National Laboratory (ORNL), and was produced at the National Renewable Energy Laboratory (NREL) from corn stover hydrolysate using a *Zymomonas mobilis* strain according to the procedures described in previous work.[26] The received broth was prefiltered with a 0.2 µm pore size polyethersulfone (PES) ultrafiltration membrane and then with a PES nanofiltration membrane (NP010, Microdyn Nadir) to ensure removal of cells, insoluble solids, and any macromolecules. The filtered broth is neutralized to pH~7 with NaOH before adsorption to dissociate organic acids into carboxylates so that they cannot be retained by hydrophobic ZIF adsorbents.

ZIF Materials synthesis

While multiple synthesis procedures for ZIF-8 and ZIF-71 are available in the literature, in this study we used the specific procedures documented in two previous works[23,25] without any modifications.

Material characterization

Freshly-made and aged materials are characterized by powder X-ray diffraction (PXRD), nitrogen physisorption, and scanning electron microscope (SEM) after activation at 180 °C under reduced pressure. PXRD measurements are performed on an X'Pert Pro PANalytical X-ray diffractometer in reflection (Bragg−Brentano) geometry operating with a Cu anode at 45 kV and 40 mA. The peak intensities are normalized with respect to the highest-intensity peak for the freshly-made and aged materials. Surface area and pore volume/size are calculated from nitrogen physisorption isotherms collected at 77 K using a BET surface area analyzer (BELSORP-max, Microtrac). SEM images were taken on a Hitachi SU8000. X-ray photoelectron spectroscopy (XPS, Thermo K-Alpha) is employed to investigate the atomic percentage of selected elements (i.e., zinc, oxygen, nitrogen, carbon, chloride) of the ZIF materials. Water vapor adsorption experiments were performed using a dynamic vapor sorption device DVS-Advantage (Surface Measurement Systems). All samples were pretreated at 100 °C for 6 h under nitrogen flow and cooled to 30 °C. Then the samples were equilibrated to water vapor at various partial pressures between 0 to 95% relative humidity.

Pelletization and adsorbent column preparation

The activated ZIF materials are pelletized using a hydraulic press under 1000 psi for 60 seconds, then gently ground and sieved to recover pellets in the 0.425-0.6 mm size range. The obtained pellets are packed into 5 cm (L) × 0.46 cm (ID) stainless steel columns.

Adsorption measurements and chemical analysis

The breakthrough experiments are performed at 303 K with a flow rate of 0.2 mL/min (superficial flow velocity of 1.2 cm/min). The model feed mixture contains representative sugars, glycerol, acetoin, and 2,3-BDO in water. The actual pretreated broth contains a larger set of

components (see Table 2). Ethanol is used as the desorbent due to its good miscibility with water and 2,3-BDO and its ease of recovery (due to high volatility). Typically, prior to breakthrough measurements, the packed bed column is regenerated in situ with ethanol at 303 K for 360 min. The outlet samples are collected periodically and analyzed via gas chromatography (GC) and high-performance liquid chromatography (HPLC). Water and ethanol are quantified by GC (Shimadzu) equipped with a Phenomenex ZB-1 column and thermal conductivity detector (TCD). The concentrations of 2,3-BDO, acetoin, xylitol, glycerol, sugars (maltose, glucose, xylose, arabinose) and organic acids (malic acid, lactic acid, acetic acid) are measured by HPLC (Shimadzu) equipped with a refractive index detector (RID) and an Aminex HPX-87H column (300 mm × 7.8 mm, Bio-Rad) at 65 °C. The mobile phase is 5 mM H_2SO_4 with a flow rate of 0.5 mL/min. The sample injection volume is 5 μL. Breakthrough curves are generated based on the outlet sample analysis as a function of time. The uptakes, q_i (mg/g or g/kg adsorbent), of each species are calculated by:

$$q_{\mathrm{i}}=\frac{C_{\mathrm{i},0}\dot{v}}{m}\int_0^t\left(\frac{C_{\mathrm{tracer,out}}}{C_{\mathrm{tracer},0}}-\frac{C_{\mathrm{i,out}}}{C_{\mathrm{i},0}}\right)\mathrm{d}t \tag{S1}$$

Here m is the mass of the adsorbent in the column (g), $\dot{v}$ is the volumetric flow rate of the feed stream (0.2 mL/min here), $C_{\mathrm{i},0}$ and $C_{\mathrm{i,out}}$ are the measured concentrations of the species i at the column inlet and outlet respectively (g/L), t is the time (min) measured from the start of the feed flow. The non-adsorbing internal tracer is glucose in the model broth, and maltose (already present) in actual broth. The separation factor for 2,3-BDO/water pair, $S_{\mathrm{2,3\text{-}BDO/water}}$, is calculated by:

$$S_{\mathrm{2,3\text{-}BDO/water}}=\frac{q_{\mathrm{2,3\text{-}BDO}}}{q_{\mathrm{water}}}\Big/\frac{C_{\mathrm{2,3\text{-}BDO,\ feed}}}{C_{\mathrm{water,\ feed}}} \tag{S2}$$

Supporting Tables

Table S1. The hydrophobicity properties and molecular size of the main components in the model and actual broths. The hydrophobicity is indicated by the *n*-octanol/water partition coefficient (log K_{ow}) and the molecular size is represented by the kinetic diameter (KD). *Maltose and glucose are considered as tracers in the adsorption measurements due to their large sizes and resulting negligible adsorption in ZIF-8 and ZIF-71. **Ethanol is included since it is used as the desorbent.

**Components**	**Formula**	**log K_{OW}**	**KD (Å)**
Maltose*	$C_{12}H_{22}O_{11}$	-5.12	11.1
Glucose*	$C_6H_{12}O_6$	-3.24	8.5
Xylose	$C_5H_{10}O_5$	-3.02	7.9
Arabinose	$C_5H_{10}O_5$	-1.98	8.1
Xylitol	$C_5H_{12}O_5$	-2.56	6.7
Glycerol	$C_3H_8O_3$	-1.76	6.3
Malic acid	$C_4H_6O_5$	-1.26	8.6
Lactic acid	$C_3H_6O_3$	-0.72	6.1
Acetic acid	$C_2H_4O_2$	-0.17	5.5
Acetoin	$C_4H_8O_2$	-0.36	5.5
2,3-BDO	$C_4H_{10}O_2$	-0.92	6.8
Water	H_2O	-1.38	2.9
Ethanol**	C_2H_6O	-0.31	4.7

Table S2. Textural properties of the ZIF-8 and ZIF-71 materials obtained from N_2 physisorption isotherms (**Figure S3**).

		BET surface area (m^2/g)	Micropore Volume (cm^3/g)
**ZIF-8**	Freshly made	1555	0.59
	Aged	1472	0.56
**ZIF-71**	Freshly made	855	0.31
	Aged	932	0.35

Table S3. The atomic percent of elements of ZIF-8 and ZIF-71 materials obtained from the XPS survey spectrum (**Figure S4**).

		Element (Atomic %)				
		Zn 2p	O 1s	N 1s	C 1s	Cl 2p
**ZIF-8**	Freshly made	8.1	2.2	31.1	58.6	N/A
	Aged	6.8	13.5	24.9	54.7	N/A
**ZIF-71**	Freshly made	6.3	6.9	20.6	42.0	24.1
	Aged	6.6	7.6	24.0	40.5	21.3

Supporting Figures

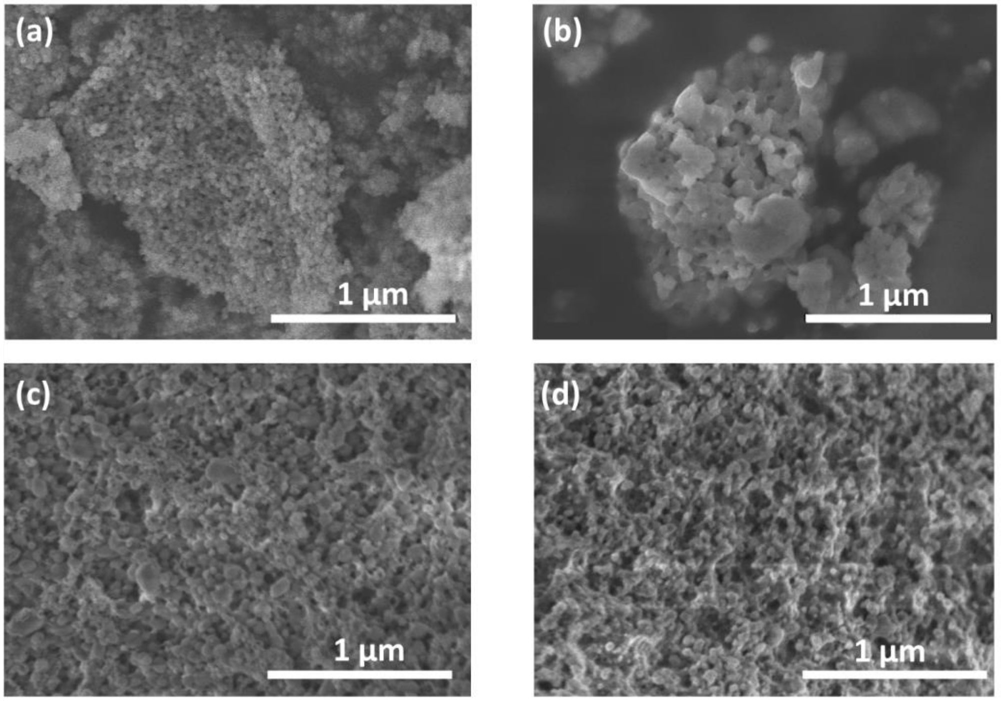

Figure S1. SEM images of newly synthesized (a) ZIF-8, (b) ZIF-71, and aged (c) ZIF-8, (d) ZIF-71.

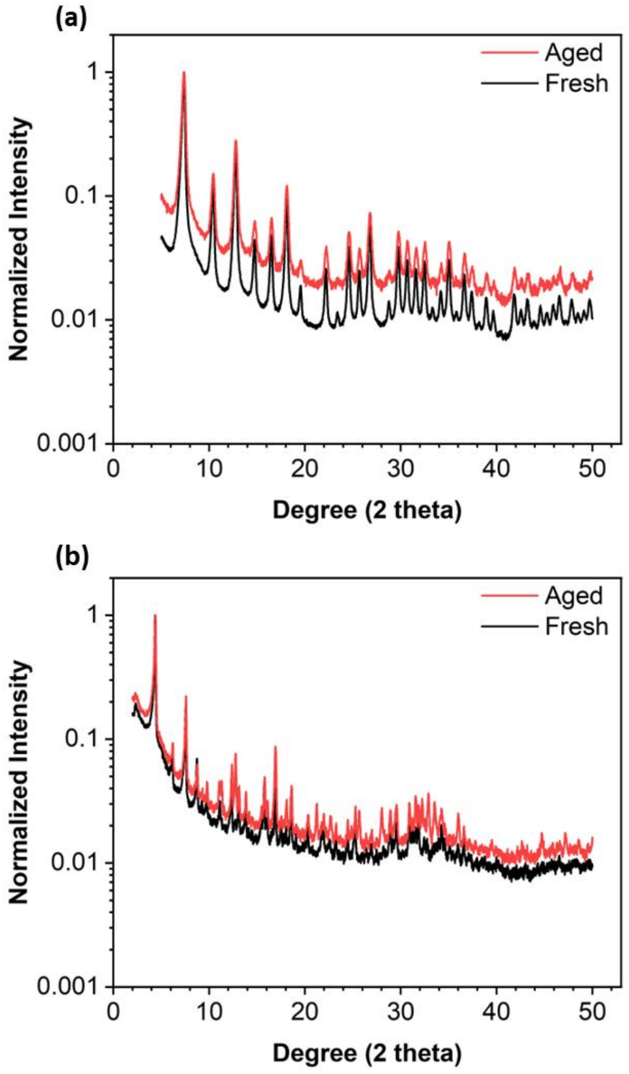

Figure S2. XRD patterns of (a) ZIF-8, (b) ZIF-71. Black curve: freshly made; Red curve: aged.

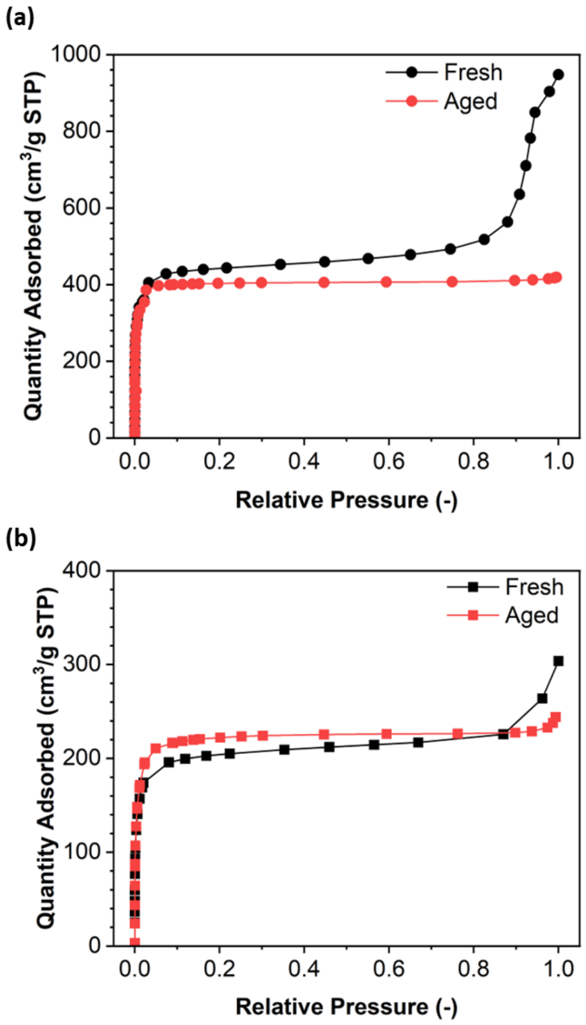

Figure S3. N_2 physisorption isotherms of (a) ZIF-8, (b) ZIF-71. Black curve: freshly made; Red curve: aged.

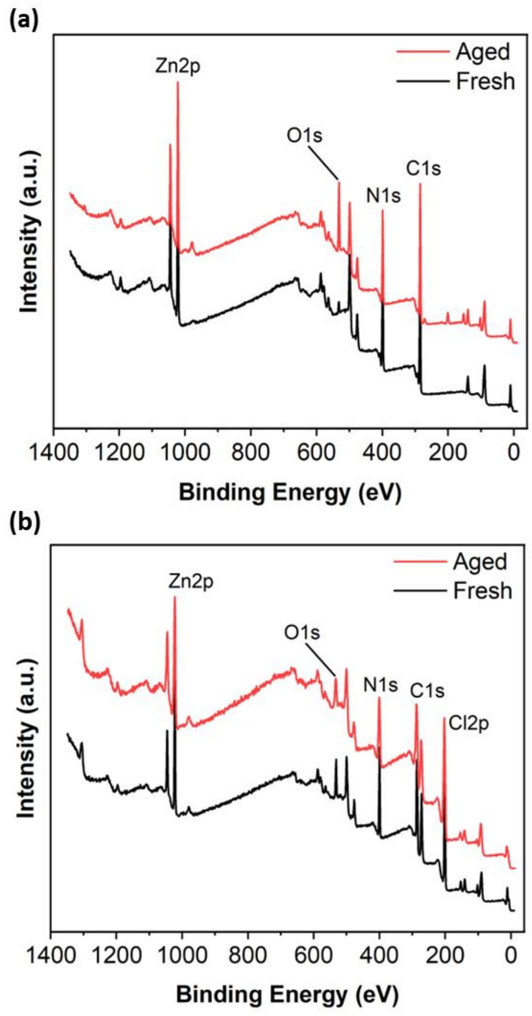

Figure S4. XPS survey spectrum of (a) ZIF-8, (b) ZIF-71. Black curve: freshly made; Red curve: aged.

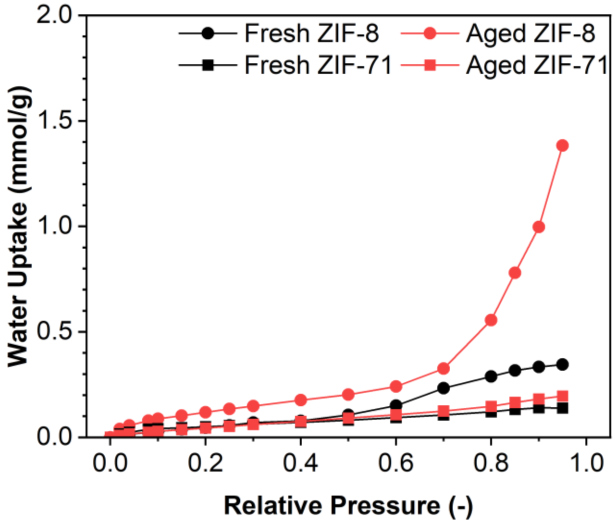

Figure S5. Water adsorption isotherms on ZIF-8 and ZIF-71 materials. Black curve: freshly made; Red curve: aged.

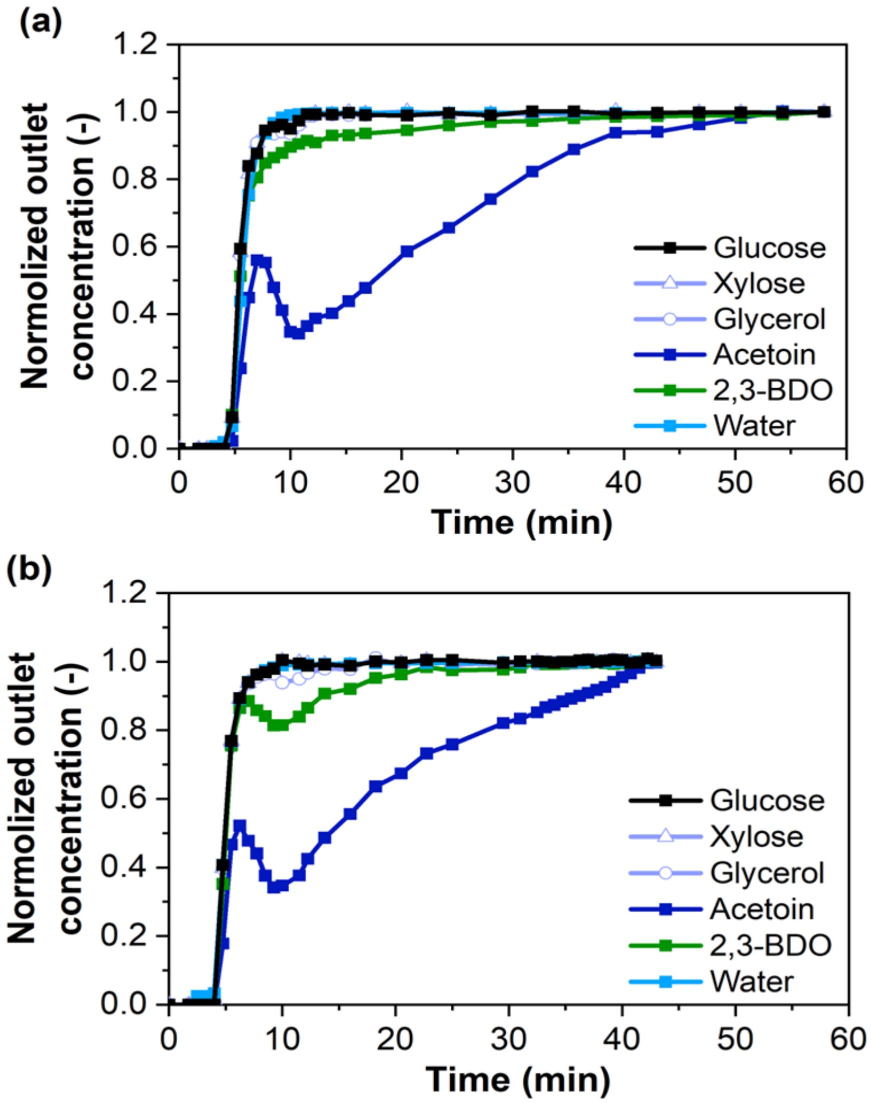

Figure S6. Adsorption breakthrough curves using model broth on the newly packed columns with freshly-synthesized (a) ZIF-8 and (b) ZIF-71 adsorbents.
